# 2-(3,4-Dimethyl-5,5-dioxo-2*H*,4*H*-pyrazolo­[4,3-*c*][1,2]benzothia­zin-2-yl)-*N*′-(3-meth­oxy­benzyl­idene)aceto­hydrazide dimethyl­formamide hemisolvate

**DOI:** 10.1107/S1600536810052177

**Published:** 2010-12-24

**Authors:** Matloob Ahmad, Hamid Latif Siddiqui, Manzoor Iqbal Khattak, Saeed Ahmad, Masood Parvez

**Affiliations:** a Institute of Chemistry, University of the Punjab, Lahore 54590, Pakistan; b Applied Chemistry Research Centre, PCSIR Laboratories Complex, Lahore 54600, Pakistan; c Department of Chemistry, University of Baluchistan, Quetta 6700, Pakistan; d Department of Chemistry, Gomal University, Dera Ismail Khan, Pakistan; e Department of Chemistry, The University of Calgary, 2500 University Drive NW, Calgary, Alberta, Canada T2N 1N4

## Abstract

In the title compound, C_21_H_21_N_5_O_4_S·0.5C_3_H_7_NO, the heterocyclic thia­zine ring adopts a half-chair conformation, with the S and N atoms displaced by −0.451 (5) and 0.233 (5) Å, respectively, from the plane formed by the remaining ring atoms. The asymmetric unit contains a disordered half-mol­ecule of solvent lying close to inversion centers. The crystal structure is stabilized by weak inter­molecular N—H⋯O and C—H⋯O inter­actions.

## Related literature

For related structures, see: Ahmad *et al.* (2008 ▶; 2009 ▶, 2011 ▶); Siddiqui *et al.* (2008 ▶). For puckering parameters, see: Cremer & Pople (1975 ▶).
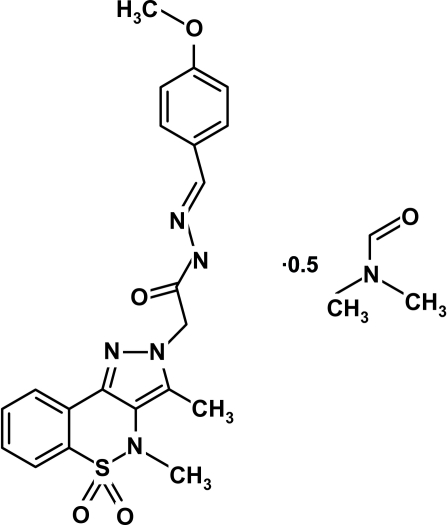


## Experimental

### 

#### Crystal data

C_21_H_21_N_5_O_4_S·0.5C_3_H_7_NO
*M* *_r_* = 476.04
Orthorhombic, 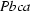

*a* = 18.3806 (5) Å
*b* = 8.1155 (2) Å
*c* = 30.4715 (5) Å
*V* = 4545.37 (18) Å^3^
*Z* = 8
Mo *K*α radiation
μ = 0.19 mm^−1^
*T* = 173 K
0.16 × 0.14 × 0.06 mm

#### Data collection

Nonius KappaCCD diffractometer
Absorption correction: multi-scan (*SORTAV*; Blessing, 1997 ▶) *T*_min_ = 0.971, *T*_max_ = 0.989
7438 measured reflections
3997 independent reflections
2747 reflections with *I* > 2σ(*I*)
*R*_int_ = 0.052

#### Refinement

*R*[*F* ^2^ > 2σ(*F* ^2^)] = 0.056
*wR*(*F* ^2^) = 0.125
*S* = 1.09
3997 reflections
328 parameters
35 restraints
H-atom parameters constrained
Δρ_max_ = 0.21 e Å^−3^
Δρ_min_ = −0.34 e Å^−3^

### 

Data collection: *COLLECT* (Hooft, 1998 ▶); cell refinement: *DENZO* (Otwinowski & Minor, 1997 ▶); data reduction: *SCALEPACK* (Otwinowski & Minor, 1997 ▶); program(s) used to solve structure: *SHELXS97* (Sheldrick, 2008 ▶); program(s) used to refine structure: *SHELXL97* (Sheldrick, 2008 ▶); molecular graphics: *ORTEP-3* for Windows (Farrugia, 1997 ▶); software used to prepare material for publication: *SHELXL97*.

## Supplementary Material

Crystal structure: contains datablocks global, I. DOI: 10.1107/S1600536810052177/jh2242sup1.cif

Structure factors: contains datablocks I. DOI: 10.1107/S1600536810052177/jh2242Isup2.hkl

Additional supplementary materials:  crystallographic information; 3D view; checkCIF report

## Figures and Tables

**Table 1:** Hydrogen-bond geometry (Å, °)

*D*—H⋯*A*	*D*—H	H⋯*A*	*D*⋯*A*	*D*—H⋯*A*
N4—H4*N*⋯O3^i^	0.88	2.06	2.878 (3)	155
C14—H14⋯O5^i^	0.95	2.49	3.287 (10)	142
C16—H16⋯O5^i^	0.95	2.35	3.145 (11)	140
C21—H21*C*⋯O2^ii^	0.98	2.53	3.497 (5)	169

Symmetry codes: (i) 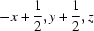; (ii) 
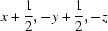
.

**Fig. 1.**
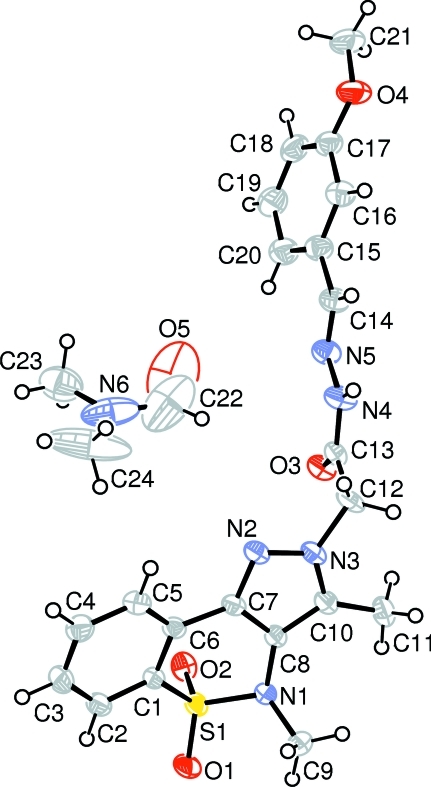
The title molecule plotted with the displacement ellipsoids at 50% probability level (Farrugia, 1997).

**Fig. 2.**
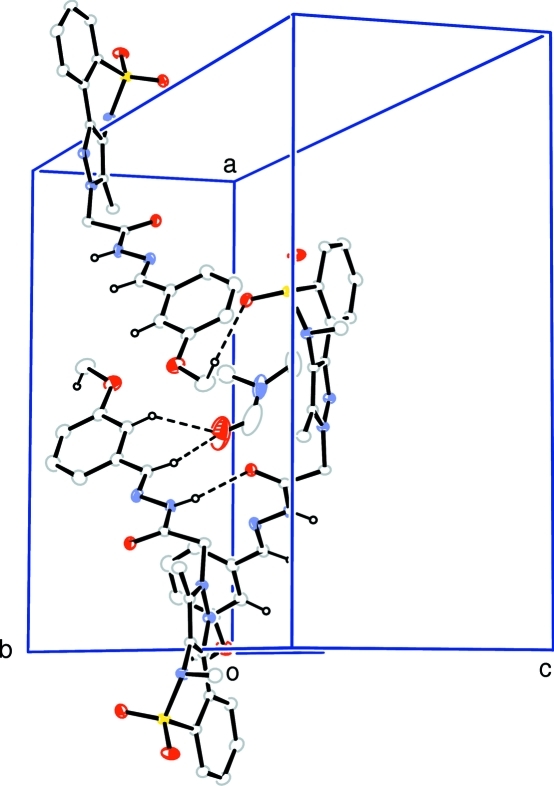
A partial packing diagram of the unit cell showing intermolecular hydrogen bonding interactions; H-atoms not involved in H-bonds have been excluded for clarity.

## supplementary crystallographic information

### Comment

In continuation to our research exploring potential biologically active derivatives of benzothiazines (Ahmad *et al.*, 2008; 2009), we have devised the fusion of the pyrazole moiety with 1,2-benzothiazine nucleus in an attempt to synthesize novel bioactive molecules. In this paper, we report the synthesis and crystal structure of the title compound, (I).

In the title molecule (Fig. 1), the heterocyclic thiazine ring adopts a half-chair conformation, with atoms S1 and N1 displaced from the plane formed by atoms C1/C6/C7/C8 by -0.451 (5) and 0.233 (5) Å, respectively. The pertinent puckering parameters (Cremer & Pople, 1975) are: *Q* = 0.445 (2) Å, θ = 61.8 (4)° and φ = 20.6 (4)°. Similar conformations of the corresponding rings have been reported in some closely related molecules (Siddiqui *et al.*, 2008; (Ahmad *et al.*, 2011). The mean-plane formed by the atoms C1–C8/C10/N2/N3 (atoms of the three fused rings excluding S1 and N1) is quite planar (maximum deviation being 0.171 (2) Å for N2) and forms an angle of 80.19 (8)° with the side chain comprised of atoms C12–C14/O3/N4/N5 which links the phenyl ring C14–C16 with the pyrazolobenzothiazin moiety; the angle between the chain atoms and the phenyl ring is 20.3 (2)°.

The intermolecular hydrogen bonds N4—H4N···O3 and C21—H21C···O1 stabilize the crystal structure. Moreover, O5 of the solvate exhibits hydrogen bonding interactions with phenyl H14 and H16 atoms (Tab. 1 and Fig. 2).

### Experimental

A mixture of 2-(3,4-dimethyl-5,5-dioxidopyrazolo[4,3-*c*][1,2]benzothiazin -2(4*H*)-yl)acetohydrazide (1.0 g, 3.12 mmol) and 3-methoxybenzaldehyde (0.42 g, 3.12 mmol) were dissolved in ethanol (50 ml) followed by the addition of 2 drops of glacial acetic acid. The mixture was subjected to reflux for 4 - 5 h. The completion of reaction was monitored with the help of thin layer chromatography (TLC). The precipitates formed were collected and washed with methanol (yield = 80%). The crystals of (I) suitable for crystallographic analysis were grown from its solution in dimethylformamide at room temperature by slow evaporation.

### Refinement

All the H atoms were discernible in the difference electron density map. However, they were positioned at the idealized positions and refined by the riding-model approximation using constraints: N—H = 0.88 Å, C—H = 0.98, 0.99 and 0.95 Å for methyl, methylene and aryl H-atoms, respectively, and *U*_iso_(H) = 1.5*U*_eq_(methyl C-atoms) and 1.2*U*_eq_(non-methyl C and N-atoms). The methyl groups were allowed to rotate about their axes during the refinement.

### Crystal data

**Table:**

C_21_H_21_N_5_O_4_S·0.5C_3_H_7_NO	*F*(000) = 2000
*M**_r_* = 476.04	*D*_x_ = 1.391 Mg m^−^^3^
Orthorhombic, *P**b**c**a*	Mo *K*α radiation, λ = 0.71073 Å
Hall symbol: -P 2ac 2ab	Cell parameters from 5739 reflections
*a* = 18.3806 (5) Å	θ = 1.0–27.5°
*b* = 8.1155 (2) Å	µ = 0.19 mm^−^^1^
*c* = 30.4715 (5) Å	*T* = 173 K
*V* = 4545.37 (18) Å^3^	Plate, colorless
*Z* = 8	0.16 × 0.14 × 0.06 mm

### Data collection

**Table:**

Nonius KappaCCD diffractometer	3997 independent reflections
Radiation source: fine-focus sealed tube	2747 reflections with *I* > 2σ(*I*)
graphite	*R*_int_ = 0.052
ω and φ scans	θ_max_ = 25.0°, θ_min_ = 1.7°
Absorption correction: multi-scan (*SORTAV*; Blessing, 1997)	*h* = −21→21
*T*_min_ = 0.971, *T*_max_ = 0.989	*k* = −9→9
7438 measured reflections	*l* = −36→36

### Refinement

**Table:**

Refinement on *F*^2^	Primary atom site location: structure-invariant direct methods
Least-squares matrix: full	Secondary atom site location: difference Fourier map
*R*[*F*^2^ > 2σ(*F*^2^)] = 0.056	Hydrogen site location: inferred from neighbouring sites
*wR*(*F*^2^) = 0.125	H-atom parameters constrained
*S* = 1.09	*w* = 1/[σ^2^(*F*_o_^2^) + (0.0337*P*)^2^ + 4.9015*P*] where *P* = (*F*_o_^2^ + 2*F*_c_^2^)/3
3997 reflections	(Δ/σ)_max_ < 0.001
328 parameters	Δρ_max_ = 0.21 e Å^−^^3^
35 restraints	Δρ_min_ = −0.34 e Å^−^^3^

### Special details

**Table:**

Experimental. *N*'-[(3-Methoxyphenyl)methylidene]-2-(3,4-dimethyl-5,5- dioxidopyrazolo[4,3-*c*][1,2]benzothiazin-1(4*H*)-yl)acetohydrazide: White powder; mp 495–496 K. IR (KBr) cm^-^1: 3449; 3364; 3033; 1692; 1616; 1310; 1164. ^1^H-NMR (DMSO-d_6_) (500 MHz) δ: 2.32 (3*H*, s, CC*H*_3_), 2.78 (3*H*, s, OC*H*_3_), 2.98 (3*H*, s, NC*H*_3_), 5.52 (2*H*, s, NC*H*_2_), 6.99–7.02 (1*H*, dd, J = 8.2, 2.0 Hz, Ar*H*), 7.26–7.38 (3*H*, m, Ar*H*), 7.63 (1*H*, t, J = 7.8 Hz, Ar*H*), 7.76 (1*H*, t, J = 7.6 Hz, Ar*H*), 7.87 (1*H*, d, J = 7.8 Hz, Ar*H*), 7.93 (1*H*, d, J = 7.7 Hz, Ar*H*), 8.03 (1*H*, s, N═C*H*), 11.79 (1*H*, br s, N*H*). ^13^C NMR: 8.5, 38.9, 47.3, 51.6, 110.5, 113.6, 117.8, 123.1, 124.1, 124.5, 126.2, 126.7, 127.5, 128.3, 130.1, 131.8, 133.4, 134.2, 136.9, 139.3, 157.6, 165.7. MS m/*z*: 439.0(*M*^+^).
Geometry. All e.s.d.'s (except the e.s.d. in the dihedral angle between two l.s. planes) are estimated using the full covariance matrix. The cell e.s.d.'s are taken into account individually in the estimation of e.s.d.'s in distances, angles and torsion angles; correlations between e.s.d.'s in cell parameters are only used when they are defined by crystal symmetry. An approximate (isotropic) treatment of cell e.s.d.'s is used for estimating e.s.d.'s involving l.s. planes.
Refinement. Refinement of *F*^2^ against ALL reflections. The weighted *R*-factor *wR* and goodness of fit *S* are based on *F*^2^, conventional *R*-factors *R* are based on *F*, with *F* set to zero for negative *F*^2^. The threshold expression of *F*^2^ > σ(*F*^2^) is used only for calculating *R*-factors(gt) *etc*. and is not relevant to the choice of reflections for refinement. *R*-factors based on *F*^2^ are statistically about twice as large as those based on *F*, and *R*- factors based on ALL data will be even larger.

### Fractional atomic coordinates and isotropic or equivalent isotropic displacement parameters (Å^2^)

**Table:**

	*x*	*y*	*z*	*U*_iso_*/*U*_eq_	Occ. (<1)
S1	−0.11871 (4)	0.35139 (10)	0.21689 (2)	0.0292 (2)	
O1	−0.17406 (11)	0.3405 (3)	0.24982 (7)	0.0414 (6)	
O2	−0.10532 (11)	0.2115 (3)	0.18941 (7)	0.0354 (5)	
O3	0.18352 (11)	0.3040 (3)	0.13232 (7)	0.0337 (5)	
O4	0.49365 (13)	0.4478 (3)	−0.07548 (7)	0.0526 (7)	
N1	−0.04176 (13)	0.3984 (3)	0.24142 (7)	0.0284 (6)	
N2	0.05545 (13)	0.6057 (3)	0.15386 (8)	0.0296 (6)	
N3	0.10980 (13)	0.5444 (3)	0.17947 (8)	0.0294 (6)	
N4	0.25112 (15)	0.5050 (3)	0.10038 (8)	0.0360 (6)	
H4N	0.2668	0.6072	0.1021	0.043*	
N5	0.27351 (14)	0.4044 (3)	0.06600 (8)	0.0355 (7)	
C1	−0.13703 (16)	0.5190 (4)	0.18165 (9)	0.0260 (7)	
C2	−0.20868 (17)	0.5616 (4)	0.17262 (10)	0.0318 (7)	
H2	−0.2475	0.5085	0.1875	0.038*	
C3	−0.22292 (18)	0.6821 (4)	0.14170 (10)	0.0372 (8)	
H3	−0.2718	0.7100	0.1348	0.045*	
C4	−0.16594 (18)	0.7623 (4)	0.12073 (10)	0.0382 (8)	
H4	−0.1761	0.8442	0.0993	0.046*	
C5	−0.09438 (17)	0.7241 (4)	0.13074 (9)	0.0329 (8)	
H5	−0.0558	0.7820	0.1169	0.040*	
C6	−0.07912 (16)	0.6004 (4)	0.16124 (9)	0.0261 (7)	
C7	−0.00550 (16)	0.5566 (4)	0.17436 (9)	0.0255 (7)	
C8	0.01144 (16)	0.4621 (4)	0.21164 (9)	0.0257 (7)	
C9	−0.04416 (17)	0.4735 (4)	0.28542 (9)	0.0341 (8)	
H9A	0.0044	0.4690	0.2987	0.051*	
H9B	−0.0787	0.4128	0.3039	0.051*	
H9C	−0.0598	0.5885	0.2830	0.051*	
C10	0.08623 (16)	0.4549 (4)	0.21424 (9)	0.0283 (7)	
C11	0.13593 (17)	0.3754 (4)	0.24619 (11)	0.0394 (8)	
H11A	0.1697	0.3027	0.2306	0.059*	
H11B	0.1074	0.3107	0.2672	0.059*	
H11C	0.1635	0.4603	0.2619	0.059*	
C12	0.18383 (16)	0.5714 (4)	0.16510 (10)	0.0350 (8)	
H12A	0.2170	0.5634	0.1906	0.042*	
H12B	0.1883	0.6836	0.1526	0.042*	
C13	0.20561 (16)	0.4462 (4)	0.13096 (9)	0.0282 (7)	
C14	0.31843 (18)	0.4724 (4)	0.03961 (10)	0.0369 (8)	
H14	0.3353	0.5807	0.0457	0.044*	
C15	0.34450 (19)	0.3878 (4)	0.00032 (10)	0.0374 (8)	
C16	0.40459 (19)	0.4517 (4)	−0.02106 (10)	0.0404 (9)	
H16	0.4265	0.5499	−0.0104	0.048*	
C17	0.43339 (18)	0.3744 (4)	−0.05791 (10)	0.0386 (8)	
C18	0.4008 (2)	0.2342 (5)	−0.07389 (10)	0.0452 (9)	
H18	0.4200	0.1807	−0.0991	0.054*	
C19	0.3398 (2)	0.1714 (5)	−0.05311 (11)	0.0507 (10)	
H19	0.3171	0.0751	−0.0645	0.061*	
C20	0.3111 (2)	0.2460 (5)	−0.01617 (11)	0.0467 (9)	
H20	0.2693	0.2016	−0.0022	0.056*	
C21	0.5293 (2)	0.3652 (6)	−0.11094 (11)	0.0629 (12)	
H21A	0.5449	0.2555	−0.1013	0.094*	
H21B	0.5719	0.4291	−0.1201	0.094*	
H21C	0.4956	0.3543	−0.1357	0.094*	
O5	0.1045 (7)	0.3207 (11)	0.0100 (4)	0.161 (5)	0.50
N6	0.0164 (11)	0.498 (3)	0.0059 (7)	0.089 (5)	0.50
C22	0.0794 (11)	0.4396 (18)	0.0258 (4)	0.130 (5)	0.50
H22	0.1006	0.4925	0.0505	0.156*	0.50
C23	−0.0119 (7)	0.401 (2)	−0.0349 (5)	0.078 (3)	0.50
H23A	−0.0556	0.4548	−0.0465	0.117*	0.50
H23B	−0.0238	0.2876	−0.0263	0.117*	0.50
H23C	0.0259	0.3988	−0.0576	0.117*	0.50
C24	−0.0275 (10)	0.6312 (17)	0.0173 (7)	0.159 (8)	0.50
H24A	−0.0674	0.6410	−0.0039	0.239*	0.50
H24B	0.0016	0.7324	0.0170	0.239*	0.50
H24C	−0.0474	0.6138	0.0467	0.239*	0.50

### Atomic displacement parameters (Å^2^)

**Table:**

	*U*^11^	*U*^22^	*U*^33^	*U*^12^	*U*^13^	*U*^23^
S1	0.0220 (4)	0.0321 (4)	0.0333 (4)	−0.0047 (4)	0.0012 (3)	0.0040 (3)
O1	0.0275 (12)	0.0564 (15)	0.0402 (12)	−0.0078 (12)	0.0080 (10)	0.0124 (11)
O2	0.0345 (13)	0.0265 (12)	0.0451 (13)	−0.0030 (10)	−0.0045 (10)	−0.0017 (10)
O3	0.0305 (12)	0.0252 (13)	0.0455 (13)	−0.0014 (10)	0.0076 (10)	−0.0047 (10)
O4	0.0473 (16)	0.0686 (19)	0.0421 (13)	0.0044 (14)	0.0185 (12)	0.0017 (13)
N1	0.0229 (14)	0.0368 (15)	0.0256 (13)	−0.0031 (12)	0.0010 (10)	0.0006 (11)
N2	0.0238 (15)	0.0303 (15)	0.0346 (14)	−0.0017 (12)	0.0051 (11)	−0.0032 (12)
N3	0.0205 (14)	0.0300 (15)	0.0377 (14)	−0.0013 (12)	0.0052 (11)	−0.0047 (12)
N4	0.0389 (16)	0.0227 (14)	0.0463 (15)	−0.0037 (13)	0.0149 (13)	−0.0102 (12)
N5	0.0354 (17)	0.0310 (16)	0.0400 (15)	0.0041 (13)	0.0079 (12)	−0.0083 (12)
C1	0.0223 (17)	0.0307 (18)	0.0249 (15)	0.0032 (14)	0.0007 (12)	−0.0020 (13)
C2	0.0256 (18)	0.0358 (19)	0.0338 (17)	0.0008 (15)	0.0040 (13)	−0.0047 (15)
C3	0.0298 (18)	0.041 (2)	0.0408 (19)	0.0104 (16)	−0.0015 (14)	−0.0012 (16)
C4	0.042 (2)	0.039 (2)	0.0342 (18)	0.0098 (17)	0.0012 (15)	0.0057 (15)
C5	0.0327 (19)	0.0325 (19)	0.0336 (17)	0.0010 (16)	0.0063 (14)	0.0010 (14)
C6	0.0244 (17)	0.0272 (17)	0.0267 (16)	0.0011 (14)	0.0029 (12)	−0.0050 (13)
C7	0.0222 (17)	0.0253 (17)	0.0289 (16)	−0.0019 (14)	0.0048 (12)	−0.0049 (13)
C8	0.0225 (17)	0.0266 (17)	0.0279 (16)	−0.0011 (14)	0.0009 (12)	−0.0007 (13)
C9	0.037 (2)	0.0392 (19)	0.0262 (16)	0.0007 (16)	0.0005 (14)	0.0034 (14)
C10	0.0238 (17)	0.0263 (17)	0.0348 (17)	−0.0010 (14)	0.0018 (13)	−0.0068 (14)
C11	0.0276 (18)	0.042 (2)	0.0482 (19)	0.0034 (17)	−0.0077 (14)	−0.0043 (17)
C12	0.0200 (18)	0.0377 (19)	0.0474 (19)	−0.0050 (15)	0.0113 (14)	−0.0116 (15)
C13	0.0201 (17)	0.0284 (18)	0.0362 (17)	−0.0007 (14)	0.0000 (13)	−0.0033 (14)
C14	0.039 (2)	0.0326 (19)	0.0396 (19)	0.0006 (17)	0.0089 (15)	−0.0040 (15)
C15	0.038 (2)	0.037 (2)	0.0371 (18)	0.0060 (17)	0.0049 (15)	0.0006 (15)
C16	0.041 (2)	0.040 (2)	0.0399 (19)	0.0023 (17)	0.0062 (16)	0.0011 (16)
C17	0.039 (2)	0.045 (2)	0.0317 (18)	0.0109 (18)	0.0045 (15)	0.0038 (16)
C18	0.050 (2)	0.055 (2)	0.0301 (18)	0.012 (2)	−0.0006 (16)	−0.0068 (17)
C19	0.056 (3)	0.056 (3)	0.040 (2)	−0.004 (2)	−0.0008 (18)	−0.0129 (18)
C20	0.046 (2)	0.054 (2)	0.040 (2)	−0.006 (2)	0.0046 (16)	−0.0066 (18)
C21	0.056 (3)	0.094 (3)	0.038 (2)	0.013 (3)	0.0173 (18)	−0.002 (2)
O5	0.238 (13)	0.067 (6)	0.178 (10)	−0.008 (7)	−0.069 (9)	0.021 (6)
N6	0.149 (15)	0.046 (4)	0.071 (10)	−0.037 (9)	0.058 (8)	−0.009 (6)
C22	0.226 (16)	0.069 (8)	0.094 (9)	−0.058 (9)	−0.033 (8)	0.025 (7)
C23	0.077 (9)	0.067 (7)	0.090 (8)	0.001 (6)	0.021 (6)	0.011 (5)
C24	0.207 (19)	0.052 (9)	0.22 (2)	−0.043 (9)	0.168 (16)	−0.024 (11)

### Geometric parameters (Å, °)

**Table:**

S1—O1	1.432 (2)	C10—C11	1.483 (4)
S1—O2	1.432 (2)	C11—H11A	0.9800
S1—N1	1.645 (2)	C11—H11B	0.9800
S1—C1	1.765 (3)	C11—H11C	0.9800
O3—C13	1.224 (4)	C12—C13	1.508 (4)
O4—C17	1.367 (4)	C12—H12A	0.9900
O4—C21	1.431 (4)	C12—H12B	0.9900
N1—C8	1.431 (4)	C14—C15	1.461 (4)
N1—C9	1.473 (4)	C14—H14	0.9500
N2—C7	1.343 (4)	C15—C16	1.383 (5)
N2—N3	1.362 (3)	C15—C20	1.398 (5)
N3—C10	1.356 (4)	C16—C17	1.391 (4)
N3—C12	1.446 (4)	C16—H16	0.9500
N4—C13	1.340 (4)	C17—C18	1.375 (5)
N4—N5	1.390 (3)	C18—C19	1.386 (5)
N4—H4N	0.8800	C18—H18	0.9500
N5—C14	1.278 (4)	C19—C20	1.382 (5)
C1—C2	1.389 (4)	C19—H19	0.9500
C1—C6	1.399 (4)	C20—H20	0.9500
C2—C3	1.383 (4)	C21—H21A	0.9800
C2—H2	0.9500	C21—H21B	0.9800
C3—C4	1.389 (5)	C21—H21C	0.9800
C3—H3	0.9500	O5—C22	1.173 (18)
C4—C5	1.385 (4)	N6—C22	1.39 (2)
C4—H4	0.9500	N6—C24	1.39 (3)
C5—C6	1.397 (4)	N6—C23	1.56 (3)
C5—H5	0.9500	C22—H22	0.9500
C6—C7	1.455 (4)	C23—H23A	0.9800
C7—C8	1.405 (4)	C23—H23B	0.9800
C8—C10	1.378 (4)	C23—H23C	0.9800
C9—H9A	0.9800	C24—H24A	0.9800
C9—H9B	0.9800	C24—H24B	0.9800
C9—H9C	0.9800	C24—H24C	0.9800
			
O1—S1—O2	118.88 (14)	H11B—C11—H11C	109.5
O1—S1—N1	107.87 (13)	N3—C12—C13	110.9 (3)
O2—S1—N1	107.57 (13)	N3—C12—H12A	109.5
O1—S1—C1	109.78 (14)	C13—C12—H12A	109.5
O2—S1—C1	106.74 (13)	N3—C12—H12B	109.5
N1—S1—C1	105.18 (13)	C13—C12—H12B	109.5
C17—O4—C21	117.6 (3)	H12A—C12—H12B	108.0
C8—N1—C9	116.6 (2)	O3—C13—N4	124.5 (3)
C8—N1—S1	112.53 (18)	O3—C13—C12	121.6 (3)
C9—N1—S1	118.9 (2)	N4—C13—C12	113.9 (3)
C7—N2—N3	103.7 (2)	N5—C14—C15	121.6 (3)
C10—N3—N2	114.1 (2)	N5—C14—H14	119.2
C10—N3—C12	128.2 (3)	C15—C14—H14	119.2
N2—N3—C12	117.5 (2)	C16—C15—C20	119.3 (3)
C13—N4—N5	120.0 (3)	C16—C15—C14	118.2 (3)
C13—N4—H4N	120.0	C20—C15—C14	122.5 (3)
N5—N4—H4N	120.0	C15—C16—C17	121.0 (3)
C14—N5—N4	114.3 (3)	C15—C16—H16	119.5
C2—C1—C6	121.1 (3)	C17—C16—H16	119.5
C2—C1—S1	119.5 (2)	O4—C17—C18	125.1 (3)
C6—C1—S1	119.3 (2)	O4—C17—C16	115.3 (3)
C3—C2—C1	119.4 (3)	C18—C17—C16	119.6 (3)
C3—C2—H2	120.3	C17—C18—C19	119.7 (3)
C1—C2—H2	120.3	C17—C18—H18	120.1
C2—C3—C4	120.1 (3)	C19—C18—H18	120.1
C2—C3—H3	119.9	C20—C19—C18	121.3 (4)
C4—C3—H3	119.9	C20—C19—H19	119.4
C5—C4—C3	120.6 (3)	C18—C19—H19	119.4
C5—C4—H4	119.7	C19—C20—C15	119.1 (3)
C3—C4—H4	119.7	C19—C20—H20	120.4
C4—C5—C6	119.9 (3)	C15—C20—H20	120.4
C4—C5—H5	120.1	O4—C21—H21A	109.5
C6—C5—H5	120.1	O4—C21—H21B	109.5
C5—C6—C1	118.9 (3)	H21A—C21—H21B	109.5
C5—C6—C7	123.0 (3)	O4—C21—H21C	109.5
C1—C6—C7	118.0 (3)	H21A—C21—H21C	109.5
N2—C7—C8	110.7 (3)	H21B—C21—H21C	109.5
N2—C7—C6	125.1 (3)	C22—N6—C24	130 (2)
C8—C7—C6	124.1 (3)	C22—N6—C23	116.9 (19)
C10—C8—C7	106.9 (3)	C24—N6—C23	113.4 (15)
C10—C8—N1	129.0 (3)	O5—C22—N6	115.5 (16)
C7—C8—N1	124.0 (3)	O5—C22—H22	122.3
N1—C9—H9A	109.5	N6—C22—H22	122.3
N1—C9—H9B	109.5	N6—C23—H23A	109.5
H9A—C9—H9B	109.5	N6—C23—H23B	109.5
N1—C9—H9C	109.5	H23A—C23—H23B	109.5
H9A—C9—H9C	109.5	N6—C23—H23C	109.5
H9B—C9—H9C	109.5	H23A—C23—H23C	109.5
N3—C10—C8	104.5 (3)	H23B—C23—H23C	109.5
N3—C10—C11	123.3 (3)	N6—C24—H24A	109.5
C8—C10—C11	132.1 (3)	N6—C24—H24B	109.5
C10—C11—H11A	109.5	H24A—C24—H24B	109.5
C10—C11—H11B	109.5	N6—C24—H24C	109.5
H11A—C11—H11B	109.5	H24A—C24—H24C	109.5
C10—C11—H11C	109.5	H24B—C24—H24C	109.5
H11A—C11—H11C	109.5		
			
O1—S1—N1—C8	−161.7 (2)	C9—N1—C8—C10	65.6 (4)
O2—S1—N1—C8	68.9 (2)	S1—N1—C8—C10	−151.9 (3)
C1—S1—N1—C8	−44.6 (2)	C9—N1—C8—C7	−110.0 (3)
O1—S1—N1—C9	−20.2 (3)	S1—N1—C8—C7	32.6 (4)
O2—S1—N1—C9	−149.5 (2)	N2—N3—C10—C8	−1.6 (3)
C1—S1—N1—C9	97.0 (2)	C12—N3—C10—C8	−176.8 (3)
C7—N2—N3—C10	2.0 (3)	N2—N3—C10—C11	179.4 (3)
C7—N2—N3—C12	177.7 (3)	C12—N3—C10—C11	4.3 (5)
C13—N4—N5—C14	−178.1 (3)	C7—C8—C10—N3	0.5 (3)
O1—S1—C1—C2	−33.0 (3)	N1—C8—C10—N3	−175.6 (3)
O2—S1—C1—C2	97.1 (2)	C7—C8—C10—C11	179.4 (3)
N1—S1—C1—C2	−148.9 (2)	N1—C8—C10—C11	3.2 (6)
O1—S1—C1—C6	150.8 (2)	C10—N3—C12—C13	93.3 (4)
O2—S1—C1—C6	−79.1 (3)	N2—N3—C12—C13	−81.7 (3)
N1—S1—C1—C6	34.9 (3)	N5—N4—C13—O3	3.3 (5)
C6—C1—C2—C3	2.5 (5)	N5—N4—C13—C12	−177.7 (3)
S1—C1—C2—C3	−173.6 (2)	N3—C12—C13—O3	−35.0 (4)
C1—C2—C3—C4	−1.6 (5)	N3—C12—C13—N4	145.9 (3)
C2—C3—C4—C5	−0.6 (5)	N4—N5—C14—C15	−176.6 (3)
C3—C4—C5—C6	1.9 (5)	N5—C14—C15—C16	−165.5 (3)
C4—C5—C6—C1	−1.0 (4)	N5—C14—C15—C20	14.5 (5)
C4—C5—C6—C7	−178.2 (3)	C20—C15—C16—C17	−1.8 (5)
C2—C1—C6—C5	−1.2 (4)	C14—C15—C16—C17	178.1 (3)
S1—C1—C6—C5	174.9 (2)	C21—O4—C17—C18	−5.6 (5)
C2—C1—C6—C7	176.1 (3)	C21—O4—C17—C16	174.5 (3)
S1—C1—C6—C7	−7.7 (4)	C15—C16—C17—O4	−178.7 (3)
N3—N2—C7—C8	−1.6 (3)	C15—C16—C17—C18	1.4 (5)
N3—N2—C7—C6	175.9 (3)	O4—C17—C18—C19	179.9 (3)
C5—C6—C7—N2	−12.6 (5)	C16—C17—C18—C19	−0.1 (5)
C1—C6—C7—N2	170.1 (3)	C17—C18—C19—C20	−0.6 (5)
C5—C6—C7—C8	164.5 (3)	C18—C19—C20—C15	0.2 (6)
C1—C6—C7—C8	−12.7 (4)	C16—C15—C20—C19	1.1 (5)
N2—C7—C8—C10	0.7 (3)	C14—C15—C20—C19	−178.9 (3)
C6—C7—C8—C10	−176.8 (3)	C24—N6—C22—O5	178 (2)
N2—C7—C8—N1	177.1 (3)	C23—N6—C22—O5	−1(2)
C6—C7—C8—N1	−0.4 (5)		

### Hydrogen-bond geometry (Å, °)

**Table:**

*D*—H···*A*	*D*—H	H···*A*	*D*···*A*	*D*—H···*A*
N4—H4N···O3^i^	0.88	2.06	2.878 (3)	155
C14—H14···O5^i^	0.95	2.49	3.287 (10)	142
C16—H16···O5^i^	0.95	2.35	3.145 (11)	140
C21—H21C···O2^ii^	0.98	2.53	3.497 (5)	169
C9—H9B···O1	0.98	2.48	2.836 (4)	101

Symmetry codes: (i) −*x*+1/2, *y*+1/2, *z*; (ii) *x*+1/2, −*y*+1/2, −*z*.
